# Predictors of extubation failure in newborns: a systematic review and meta-analysis

**DOI:** 10.1186/s13052-023-01538-0

**Published:** 2023-10-02

**Authors:** Maoling Fu, Zhenjing Hu, Genzhen Yu, Ying Luo, Xiaoju Xiong, Qiaoyue Yang, Wenshuai Song, Yaqi Yu, Ting Yang

**Affiliations:** 1 Department of Nursing, Tongji Hospital, Tongji Medical College, Huazhong University of Science and Technology, Qiaokou District, 1095 Jiefang Road, Wuhan, Hubei Province, China; 2 School of Nursing, Tongji Medical College, Huazhong University of Science and Technology, Wuhan, Hubei China

**Keywords:** Extubation failure, Newborns, Predictors, Systematic review, Meta-analysis

## Abstract

Extubation failure (EF) is a significant concern in mechanically ventilated newborns, and predicting its occurrence is an ongoing area of research. To investigate the predictors of EF in newborns undergoing planned extubation, we conducted a systematic review and meta-analysis. A systematic literature search was conducted in PubMed, Web of Science, Embase, and Cochrane Library for studies published in English from the inception of each database to March 2023. The PRISMA guidelines were followed in all phases of this systematic review. The Risk of Bias Assessment for Nonrandomized Studies tool was used to assess methodological quality. Thirty-four studies were included, 10 of which were overall low risk of bias, 15 of moderate risk of bias, and 9 of high risk of bias. The studies reported 43 possible predictors in six broad categories (intrinsic factors; maternal factors; diseases and adverse conditions of the newborn; treatment of the newborn; characteristics before and after extubation; and clinical scores and composite indicators). Through a qualitative synthesis of 43 predictors and a quantitative meta-analysis of 19 factors, we identified five definite factors, eight possible factors, and 22 unclear factors related to EF. Definite factors included gestational age, sepsis, pre-extubation pH, pre-extubation FiO_2_, and respiratory severity score. Possible factors included age at extubation, anemia, inotropic use, mean airway pressure, pre-extubation PCO_2_, mechanical ventilation duration, Apgar score, and spontaneous breathing trial. With only a few high-quality studies currently available, well-designed and more extensive prospective studies investigating the predictors affecting EF are still needed. In the future, it will be important to explore the possibility of combining multiple predictors or assessment tools to enhance the accuracy of predicting extubation outcomes in clinical practice.

**Supplementary Information:**

The online version contains supplementary material available at 10.1186/s13052-023-01538-0.

## Introduction

Invasive mechanical ventilation (MV) is a life support procedure regularly employed as an auxiliary ventilation method in intensive care [1, 2]. Mechanical ventilators have been utilized since the 1960s to support respiratory failure and improve survival in low-birth-weight preterm infants [3]. Despite the ability of endotracheal intubation and MV to save lives, they are associated with several complications, such as bacterial colonization, sepsis, ventilator-associated pneumonia, and airway trauma. Moreover, prolonged MV increases the risk of potential complications, including bronchopulmonary dysplasia (BPD), neurodevelopmental disorders, periventricular hemorrhage, subglottic stenosis, laryngeal edema, diaphragmatic atrophy, emphysema, pneumothorax and reduced postnatal growth [4–7]. Therefore, to minimize these risks and complications, it is the current consensus in clinical practice to withdraw from the ventilator as soon as possible and shorten the duration of invasive mechanical ventilation [8].

The scientific basis for determining when a patient is ready for extubation is still imprecise, despite significant advancements in MV and post-extubation respiratory support in neonatology. Clinical judgment, personal experience, bedside observation of blood gases, oxygen requirements, and ventilator settings are typically used to make decisions on whether to extubate [9, 10]. Consequently, there are significant practical differences and a paucity of protocols to simplify the management of all components of the peri-extubation process, with decisions often being physician-dependent rather than evidence-based, which may lead to inappropriate extubation [11–13]. The rate of extubation failure (EF) increases from 20% in infants born at 28–31 weeks gestational age to more than 60% in very preterm infants born at less than 28 weeks gestational age [14–17] for several reasons, including frequent or severe apneas, residual lung disease, immature respiratory drive and presence of unstable patent ductus arteriosus. EF not only prolongs the duration of mechanical ventilation but is independently associated with increased mortality, morbidity, length of hospital stay, and healthcare costs [17–19]. Therefore, it is critical and challenging to determine the optimal timing of extubation in mechanically ventilated neonates.

Identifying factors associated with failed extubation may help reduce the duration of mechanical ventilation, avoid reintubation, improve outcomes, and design future studies of ventilated preterm infants. This study aimed to identify potential predictors of EF in newborns.

## Methods

The review followed the PRISMA reporting guidelines, a 27-item list designed to improve the reporting of systematic evaluations [20], and was registered with PROSPERO (registration number: CRD42023415289). All relevant analyses were based on previously published studies and did not require ethical approval or patient consent.

### Search strategy

A systematic literature search was conducted in PubMed, Web of Science, Embase, and Cochrane Library for studies that were published in English from the inception of each database to March 2023 using keywords, Medical Subject Headings (MeSH), and other index terms, as well as combinations of these terms and appropriate synonyms. The search terms focused on “newborn infant,” “newborn,” “neonate,” “extubation failure,” “EF,” “extubation outcome,” “extubation readiness,” “risk factors,” “Influencing factors,” “predictors,” and their synonyms (see the Supplementary Material for the complete search strategy). Additionally, we manually searched the reference lists of all selected studies for any further relevant studies meeting our inclusion criteria.

### Inclusion criteria

- Newborns were the majority of the study population, including preterm infants and low-birth-weight infants (LBWI).
- Extubation failure/success as the primary outcome indicator and predictors of extubation failure/success as the primary study objective.
- Prospective or retrospective cohort study.
- Studies published in English.

### Exclusion criteria

- Adults, children, or adolescents were the majority of the study’s population.
- Focus on specific disease areas, such as congenital heart disease, laryngotracheoplasty, burns, or other surgical intubation.
- Accidental extubation, treatment failure, intubation time, or death as the primary outcome indicator.
- Clinical trial articles, because the clinical trial population cannot replace other MV newborns.
- Abstracts, clinical trial registries, and medical record reports.
- Conference proceedings, review articles, letters, and editorials.
- Animal or in vitro studies.
- Unavailable original literature.
- Not published in English.

### Data extraction

Two reviewers extracted the data using a pre-designed Microsoft Excel 2019 spreadsheet. The extraction procedure was conducted independently, with a third senior reviewer mediating disputes when necessary. Data were collected on the following characteristics for each included study.

1. Basic information: first author, country, year of publication, study duration, and study design.
2. Demographic characteristics: exclusion criteria of the study, sample size, number and rate of EF.
3. Assessment of reintubation: type of respiratory support provided after extubation and criteria for reintubation.
4. Description of EF: the primary definition and time frame used to classify infants into extubation success or failure were recorded.
5. Predictors of EF.

### Quality assessment

Two reviewers independently assessed the methodological quality of the study, and disagreements were settled by consensus through a panel discussion. The risk of bias for each included study was assessed using the Risk of Bias Assessment for Nonrandomized Studies tool [21]. This tool was selected because of the nonrandomized nature of all included studies as well as its ability to evaluate six domains of risk of bias, including 1) selection of participants, 2) confounding variables, 3) measurement of exposure, 4) blinding of outcome assessments, 5) incomplete outcome data, and 6) selective outcome reporting. If the study received low risk ratings for each of the six evaluated domains, the risk of bias would be low. If at least one domain was rated to have an unclear risk (but no domains were rated to have a high risk), the study would be at moderate risk of bias. If at least one domain was rated as having a high risk, the study would be a high risk of bias.

A third reviewer extracted data from five studies that were chosen at random and examined for methodological quality and bias risk to ensure the correctness of the assessment.

### Qualitative synthesis and quantitative meta-analysis

Each reported predictor was synthesized qualitatively based on the total number of low and moderate risk of bias studies evaluating the factor and the percentage of studies showing positive correlation, marking it as definite, likely, unclear, or not a risk factor (Table 1). For each risk factor, adjusted or unadjusted odds ratios were recorded when available. For predictors with sufficiently homogeneous definitions and reference ranges, a quantitative meta-analysis of low and moderate risk of bias studies was implemented to estimate a combined OR.

**Table 1:** Defining the strength of a risk factor

Definite
All low and moderate risk of bias studies positive (at least three studies)
Majority (more than 50%) low and moderate risk of bias studies positive (at least five studies)
Likely
All low and moderate risk of bias studies positive (two studies)
Majority (more than 50%) low and moderate risk of bias studies positive (2–4 studies)
Unclear
All low and moderate risk of bias studies positive (one study)
Low and moderate risk of bias studies show mixed or conflicting results
A majority (more than 50%) of studies negative but at least one low or moderate risk of bias study positive
Not a risk factor
No low or moderate risk of bias studies positive

Data analysis was performed using Revman5.4 software provided by the Cochrane Collaboration Network. The generic inverse variance method was used for the meta-analysis of both predictors and EF rates [22]. This method requires only effect estimates and their SEs. The SEs were estimated by back transforming the 95% confidence limits using the standard normal distribution. The included studies were tested for heterogeneity (I^2^ test), if *P* ≥ 0.05 and I^2^ < 50%, this indicated less heterogeneity among studies and a fixed-effects model was selected for statistical analysis of the data, while conversely *P* < 0.05 or I^2^ ≥ 50% indicated greater heterogeneity among studies and a random-effects model was used.

## Results

**Fig. 1:**
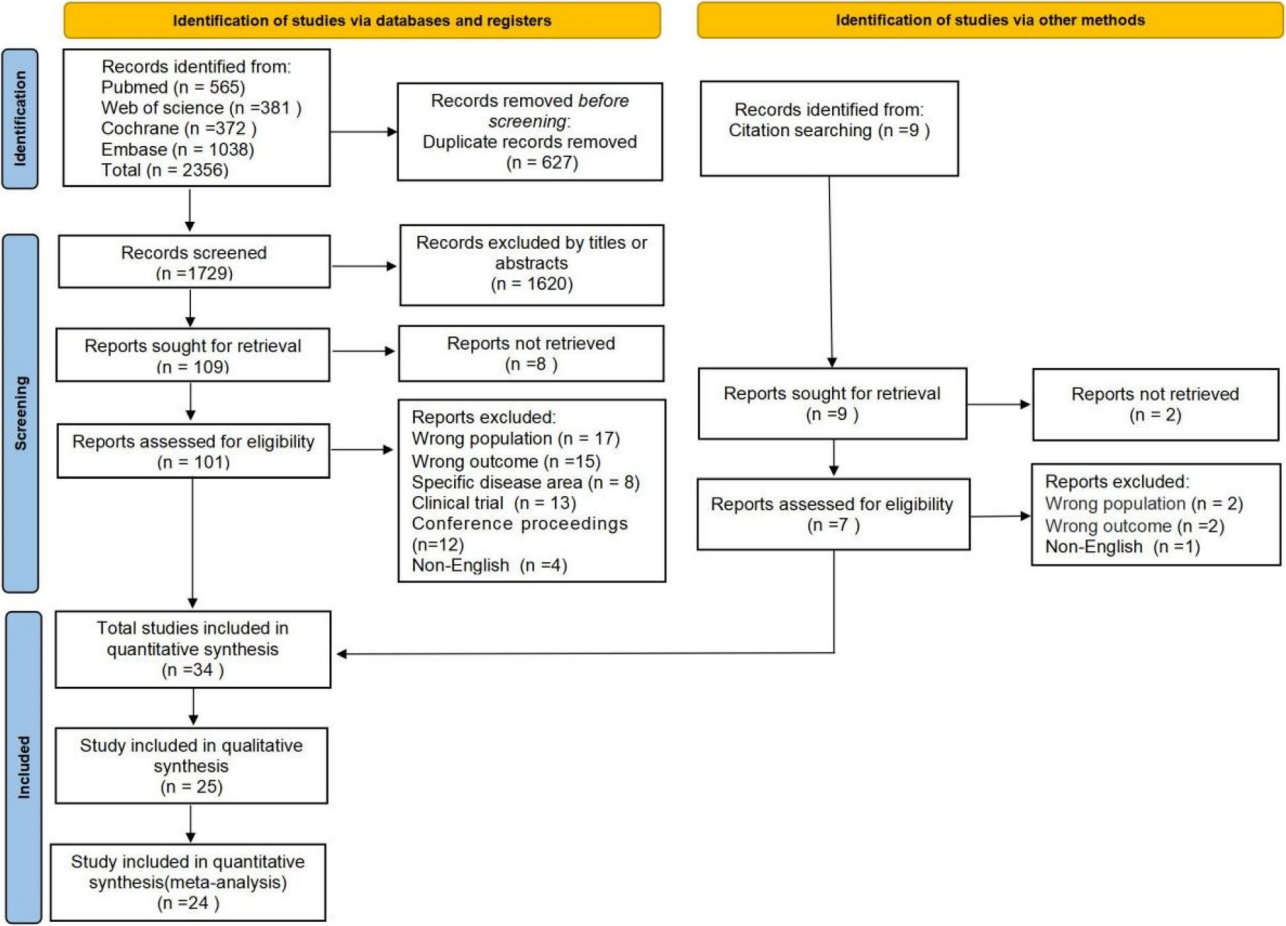
Flow chart of the systematic literature search

A total of 2356 articles were identified from the literature search of the databases, of which 627 duplicates were removed. After screening the remaining 1729 articles for title and abstract, 101 articles were selected for full-text retrieval. Following the eligibility assessment, 32 articles met the inclusion criteria. The references of the selected articles were also examined, and a full-text search was conducted for nine articles, resulting in the inclusion of two articles that met the eligibility criteria. Ultimately, 34 articles were identified for inclusion in this review, with 25 studies contributing to the qualitative synthesis and 24 to the quantitative meta-analysis. Study identification is summarized in Fig. 1.

Of these studies, 19 were prospective studies [17, 19, 23–39] and the remaining 15 studies were retrospective [10, 16, 40–52], of which seven were multicenter studies [16, 17, 19, 23, 37, 39, 49] and 27 were single-center studies [10, 24–36, 38, 40–48, 50–52].The sample size ranged from 34 to 926, with the two largest studies including 394 newborns [40] and 926 newborns [17], respectively. Three of these studies constructed clinical prediction models [16, 42, 49]. The basic characteristics of the included literature are given in Table 2.

**Table 2:** Summary characteristics of the 34 studies included in this review

Author	Country of study	Year of publication (data)	Study Design	Exclusions in study	Study population	Sample size	EF rate	Criteria for reintubation	Definition of EF	Post-extubation respiratory support	Study quality
Alaa et al [46]	Qatar	2021 (2016–2017)	Retrospective	1 congenital anomalies;2 IVH(grade III and IV)	28–36 weeks gestational age	220	18 (8.2%)	FiO_2_ > 0.35–0.40;frequent or severe apnea;blood gas pH < 7.25 and/ or PCO_2 _> 60 mmHg	reintubation within 7 days of extubation	NCPAP; NIPPV; NC	Low
Bahgat et al [30]	Egypt	2021 (2017–2019)	Prospective	1 congenital anomalies; 2 accidental extubation;3 pleural effusion	< 32 weeks gestational age	43	9 (20.9%)	FiO_2_ > 0.6;blood gas pH < 7.25and/ or PCO_2_ > 65 mmHg;frequent or severe apnea	reintubation within 72 h of extubation	NCPAP	High
Bhat et al [25]	Britain	2016 (Not mentioned)	Prospective	major congenital abnormalities	All gestations	60	12 (20.0%)	frequent or severe apnea; blood gas pH < 7.2 or failure to improve despite instituting CPAP	reintubation within 48 h of extubation	NCPAP	High
Chawla et al [33]	America	2013 (2008–2009)	Prospective	accidental extubation;	< 32 weeks gestational age	49	10 (20.4%)	increase in FiO_2_ of > 40% baseline;blood gas pH < 7.25 and/ or PCO_2_ > 60 mmHg; frequent or severe apnea	reintubation within 72 h of extubation	NCPAP;HFNC;NIPPV;NC	Low
Chawla et al [17]	America	2017 (2005–2009)	Prospective	1 congenital anomalies; 2 death before extubation; 3 accidental extubation;4 transfer	24–27 weeks gestational age	926	388 (41.9%)	FiO_2_ > 0.5;blood gas PCO_2_ > 65 mmHg; frequent apnea;clinical shock, sepsis, and/or the need for surgery	reintubation within 5 days of extubation	Not mentioned	Moderate
Chen et al [41]	China	2022 (2017–2021)	Retrospective	1 death before extubation; 2 accidental extubation; 3 congenital anomalies	≤ 1500 g birth weight	60	13 (21.7%)	Not mentioned	reintubation within 72 h of extubation	NIV or NCPAP	Moderate
Cheng et al [49]	China	2021 (2015–2020)	Retrospective	1 congenital anomalies; 2 accidental extubation; 3 death before extubation;4 missing data;5surgical	25–29 weeks gestational age	128	55 (43.0%)	FiO_2_ > 0.6–0.7;blood gas pH < 7.2and/ or PCO_2_ > 60-65 mmHg; frequent apnea; required PS treatment	reintubation within 5 days of extubation	Not mentioned	Moderate
Dassios et al [34]	Britain	2017 (2016)	Prospective	1 congenital anomalies; 2 accidental extubation	< 34 weeks gestational age	46	23 (50.0%)	FiO_2_ > 0.5;blood gas pH < 7.25 and/ or PCO_2_ > 55-60 mmHg;frequent or severe apnea	reintubation within 72 h of extubation	NCPAP;HFNC;NIPPV;	Low
Devadas et al [24]	India	2019 (2015–2017)	Prospective	1 congenital anomalies; 2 IVH(grade III and IV);3 death before extubation;4 perinatal asphyxia	> 26 weeks gestational age; > 600 g birth weight	93	41 (44.1%)	FiO_2_ ≥ 0.5; blood gas pH < 7.25 and/ or PCO_2_ > 65 mmHg; frequent or severe apnea; Downes score ≥ 6	reintubation within 48 h of extubation	NCPAP or head box oxygen	High
Dimitriou et al [27]	Britain	2002 (Not mentioned)	Prospective	Not mentioned	< 37 weeks gestational age	36	7 (19.4%)	severe apnoea; blood gas pH < 7.2; failed to improve despite the institution of CPAP	reintubation within 48 h of extubation	NCPAP or head box oxygen	Moderate
Dimitriou et al [26]	Greece	2011 (2007–2008)	Prospective	1 congenital anomalies; 2 congenital infections	< 37 weeks gestational age	56	8 (14.3%)	severe stridor and apnea with bradycardia; requiring bag ventilation; blood gas pH < 7.2 or required FiO_2_ 0.6	reintubation within 48 h of extubation	NCPAP or head box oxygen	High
Dursun et al [10]	Turkey	2021 (2016–2020)	Retrospective	1 death before extubation;2 accidental extubation; 3congenital anomalies; 4 transfer	< 30 weeks gestational age; < 1250 g birth weight	142	43 (30.2%)	blood gas pH < 7.2 and/ or PCO_2_ > 65 mmHg; failure to sustain target oxygen saturation; frequent or severe apnea	reintubation within 72 h of extubation	NCPAP or NIPPV	Low
Gupta et al [42]	America	2019 (2009–2015)	Retrospective	1 death before extubation;2 missing data	< 1250 g birth weight	312	84 (26.9%)	frequent or severe apnea; blood gas pH < 7.2 and/ or PCO_2_ > 65 mmHg	reintubation within 5 days of extubation	NCPAP; NIPPV; NC	Moderate
Hathlol et al [40]	Saudi Arabia	2017 (2000–2014)	Retrospective	1 death before extubation; 2 missing data;3chromosomal or congenital anomalies	≤ 1500 g birth weight	394	47 (11.9%)	primarily on clinical assessment, blood gas levels, and ventilatory parameters	reintubation within 72 h of extubation	NCPAP or NIMV	Moderate
He et al [43]	China	2022 (2016–2020)	Retrospective	1 congenital anomalies;2 request for discharge;3 pulmonary hypoplasia	< 32 weeks gestational age	359	110 (30.6%)	blood gas pH < 7.2 and/ or PCO_2_ > 65 mmHg;FiO > 0.6;frequent apnea; clinical shock;sepsis;surgery	reintubation within 72 h of extubation	NCPAP or NIPPV	Low
Hermeto et al [44]	Canada	2009 (2002–2004)	Retrospective	1 congenital anomalies;2 transfer;3missing data	< 1250 g birth weight	39	9 (23.1%)	blood gas pH < 7.25 and/ or PCO_2 _> 65 mmHg;frequent or severe apnea; need for oxygen higher than 50% in CPAP	reintubation within 7 days of extubation	NCPAP	Moderate
Hilal et al [45]	Oman	2022 (2013–2017)	Retrospective	1 death before extubation;2 palliative or comfort care; 3accidental extubation; 4 transfer	< 37 weeks gestational age	140	34 (24.3%)	desaturation and bradycardia;apnoea and respiratory failure	reintubation within 7 days of extubation	NCPAP;NHF; NIPPV	Moderate
Hiremath et al [28]	India	2009 (Not mentioned)	Prospective	1 congenital anomalies;2 HIE stage III or IVH grade 4	All gestations	82	22 (26.8%)	increase in FiO_2_of > 50% baseline;increase in respiratory rate of > 25% baseline; Downe’score > 6;Silverman’ score ≥ 7;PaO_2 _< 50 mmHg, or PCO_2_ > 60 mmHg	reintubation within 48 h of extubation	NCPAP or head box oxygen	Moderate
Hunt et al [31]	Britain	2020 (2016–2018)	Prospective	congenital anomalies	All gestations	72	15 (20.8%)	FiO_2_ > 0.7;blood gas pH < 7.2;frequent apnea	reintubation within 48 h of extubation	NCPAP;HFNC;incubator oxygen	High
Kaczmarek et al [23]	Canada	2013 (2010–2011)	Prospective	1 congenital anomalies; 2 congenital heart disease;3 used vasopressors/sedation	< 1250 g birth weight	47	11 (23.4%)	FiO_2_ > 0.5;blood gas pH < 7.25 and/ or PCO_2_ > 55-60 mmHg;frequent apnea; significantly increased work of breathing	reintubation within 72 h of extubation	NCPAP or NIPPV	Moderate
Kaczmarek et al [52]	Canada	2013 (2005–2006)	Retrospective	1 congenital anomalies; 2 missing data;3 death before extubation	< 1250 g birth weight	44	8 (18.2%)	FiO_2_ > 0.6;blood gas pH < 7.25 and/ or PCO_2_ > 65 mmHg;frequent or severe apnea	reintubation within 72 h of extubation	NCPAP or NIPPV	High
Kamlin et al [35]	Australia	2006 (2003–2004)	Prospective	mechanical ventilation for less than 24 h	< 1250 g birth weight	50	11 (22.0%)	FiO_2_ > 0.6;blood gas pH < 7.25 and/ or PCO_2_ > 65 mmHg;frequent or severe apnea	reintubation within 72 h of extubation	NCPAP or NIPPV	High
Kidman et al [16]	Australia	2021 (2016–2017)	Retrospective	1 death before extubation;2 accidental extubation	< 28 weeks gestational age	204	96 (47.1%)	apnoea;increasing FiO_2_ requirement; respiratory acidosis	reintubation within 7 days of extubation	NCPAP;NHF; NIPPV	Moderate
Manley et al [19]	Australia	2016 (2010–2012)	Prospective	1 congenital anomalies;2 not providing maximal intensive care	< 28 weeks gestational age	174	56 (32.2%)	increase in FiO_2_ of > 20% baseline;blood gas pH < 7.2 and/ or PCO_2_ > 60 mmHg; frequent or severe apnea; urgent need	reintubation within 7 days of extubation	NIPPV or HFNC	High
Menshykova et al [36]	Ukraine	2017 (Not mentioned)	Prospective	Not mentioned	< 1500 g birth weight	92	27 (29.3%)	FiO_2_ ≥ 0.6;blood gas pH < 7.25 and/ or PCO_2 _≥ 55 mmHg;frequent or severe apnea	reintubation within 72 h of extubation	NCPAP;HFNC;NIV	Moderate
Mhanna et al [47]	America	2017 (2009–2012)	Retrospective	1 death before extubation; 2 admission for comfort care	< 1500 g birth weight	147	45 (30.6%)	FiO_2_ > 0.5;blood gas pH < 7.2 and/ or PCO_2 _> 65 mmHg	reintubation within 48 h of extubation	NCPAP; NIMV;NC	Low
Mohsen et al [29]	Canada	2023 (2019–2021)	Prospective	1 congenital anomalies;2 pneumothorax;3 pleural effusion;4 use muscle relaxant;	< 28 weeks gestational age	45	9 (20.0%)	FiO_2_ > 0.4–0.5;blood gas pH < 7.2 and/ or PCO_2_ > 65 mmHg; frequent or severe apnea	reintubation within 72 h of extubation	NIPPV	Low
Ohnstad et al [39]	Norway	2022 (2013–2018)	Prospective	1 death before extubation; 2 accidental extubation; 3 missing data	< 26 weeks gestational age	316	143 (45.3%)	Not mentioned	reintubation within 72 h of extubation	NCPAP or BiPAP	High
Shalish et al [37]	Canada	2020 (2013–2018)	Prospective	1 congenital anomalies; 2 death before extubation; 3 accidental extubation;; 4 used vasopressors/sedation	< 1250 g birth weight	259	75 (29.0%)	FiO_2 _> 0.6;blood gas pH < 7.25 and/ or PCO_2_ > 65 mmHg;frequent or severe apnea	reintubation within 7 days of extubation	NCPAP or NIPPV	Moderate
Spaggiari et al [50]	Italy	2022 (2010–2019)	Retrospective	1 congenital anomalies;2 accidental extubation;3 intubation after the first 24 h of life	< 28 weeks gestational age; < 1000 g birth weight	80	29 (36.2%)	blood gas pH < 7.2 and/ or PCO_2_ > 65 mmHg;frequent apnea	reintubation within 72 h of extubation	NCPAP	Low
Su et al [48]	Korea	2023 (2017–2021)	Retrospective	1 death before extubation; 2 transfer	< 32 weeks gestational age	129	24 (18.6%)	FiO_2_ above the initial value;SARS > 4;frequent or severe apnea;blood gas pH < 7.2 and/ or PCO_2_ > 65 mmHg	reintubation within 7 days of extubation	NCPAP or HFNC	Low
Teixeira et al [32]	Brazil	2020 (2018–2019)	Prospective	1 congenital anomalies; 2 death before extubation; 3 missing data	< 1500 g birth weight	112	26 (23.2%)	FiO_2_ > 0.5;blood gas pH < 7.2 and/ or PCO_2_ > 55-60 mmHg; frequent apnea; significantly increased work of breathing	reintubation within 7 days of extubation	NCPAP or NIPPV	Moderate
Wang et al [51]	China	2017 (2009–2013)	Retrospective	1 death before extubation;2 congenital anomalies	< 1000 g birth weight	68	16 (23.5%)	FiO_2_ > 0.6;blood gas pH < 7.25 and/ or PCO_2_ > 65 mmHg; frequent apnea; significantly increased work of breathing	reintubation within 7 days of extubation	NCPAP or NIPPV	Low
Williams et al [38]	Britain	2022 (2020–2021)	Prospective	congenital lung or diaphragmatic anomalies	< 37 weeks gestational age	48	13 (27.1%)	FiO_2_ > 0.6;blood gas pH < 7.25 and/ or PCO_2_ > 65 mmHg;severe apnea	reintubation within 48 h of extubation	NCPAP or HFNC	Moderate

### Study populations and EF rates in included studies

The studies investigated a range of newborns of different gestational ages and different birth weights, of which 16 studies were conducted with preterm infants as the study population; 12 studies were conducted with LBWI as the study population; three studies with different requirements for gestational age or birth weight; and the other three studies included all eligible newborns without other requirements for gestational age or birth weight.

The combined EF rate was 26.5% (95% confidence interval [CI]: 23.1–30.6%). The heterogeneity was high (I^2^ = 88%). The combined EF rate was 26.5% (95% CI: 21.9–31.5%; I^2^ = 77%) in female infants and 32.9%(95% CI: 28.1–37.5%; I^2^ = 78%) in male infants. The frequency of EF based on very low birth weight (VLBW) infants and extremely preterm infants were available from 12 articles with 1698 infants and 7 articles with 1873 infants. The combined EF rate with VLBW infants and extremely preterm infants were 24.2% (95% CI: 20.0–28.6%; I^2^ = 75%), 40.1% (95% CI: 35.5–44.8%; I^2^ = 69%), respectively (Fig. S1-5 in the supplementary material).

### Definition of EF and Criteria for reintubation

There was heterogeneity in the definition of EF among the included studies (Table 2). In all studies, EF was defined as reintubation, but the time range used to define EF ranged from 48 h to 7 days. Of these, eight studies defined EF as reintubation within 48 h with a combined EF rate of 25.9% (95% CI: 20.0–32.9%; I^2^ = 68%), 14 studies defined EF as reintubation within 72 h with a combined EF rate of 26.5% (95% CI: 20.6–33.8%; I^2^ = 88%), three studies defined EF as reintubation within 5 days with a combined EF rate of 36.7% (95% CI: 27.0–47.9%; I^2^ = 91%), and nine studies defined EF as reintubation within 7 days with a combined EF rate of 24.2% (95% CI: 17.4–32.9%; I^2^ = 90%) (Fig. S6 in the supplementary material).

There is no uniform standard regarding reintubation criteria. In studies proposing indications for reintubation, the most commonly used index is inspired oxygen fraction (FiO_2_) > 0.5–0.7; partial pressure of carbon dioxide (PCO_2_) > 55–65 mmHg, with persistent acidosis (pH < 7.20–7.25); frequent or severe apnea; increased work of breathing.

### Quality of the EF studies

**Fig. 2:**
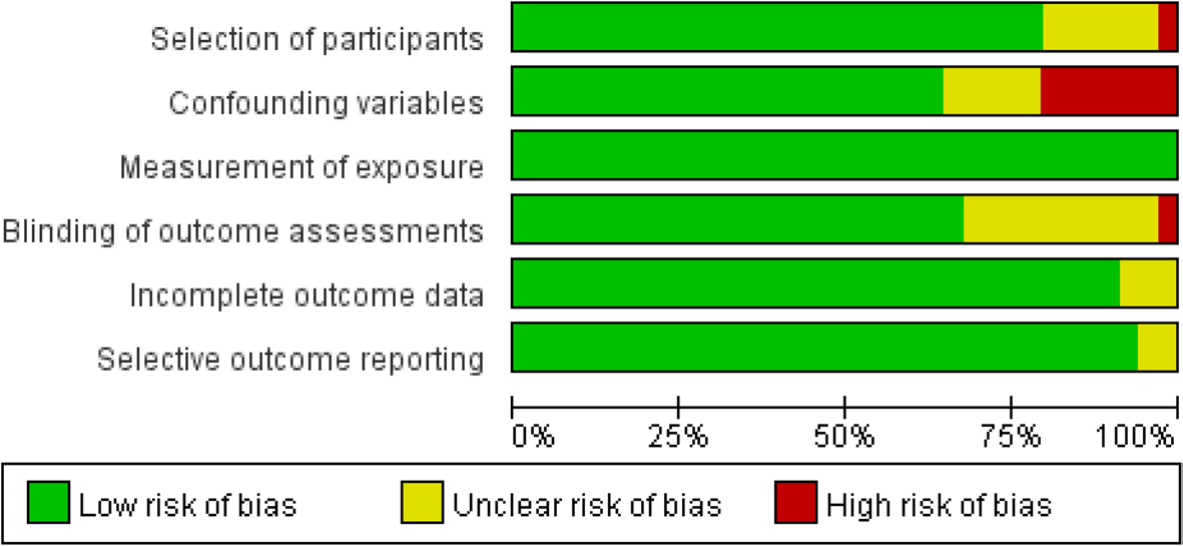
Summary of risk of bias in the included studies

Included studies differed in their methodological quality (Fig. 2, and Fig. S7 in the supplementary material). Ten studies were classified as low risk in all six domains and were considered to be at overall low risk of bias. Nine studies were considered to be at overall high risk of bias, of which seven were related to confounding variables, one was related to selection of participants, and one was related to outcome assessments. The remaining 15 studies had at least one unclear risk in six domains and were categorized as having a moderate risk of bias.

### Predictors of EF in included studies

The 34 included studies described 43 statistically significant risk factors for EF. These variables were divided into six major categories: intrinsic factors (47.1%, 16/34); maternal factors (11.8%, 4/34); diseases and adverse conditions of the newborn (17.6%, 6/34); treatment of the newborn (8.9%, 3/34); characteristics before and after extubation (38.2%, 13/34); and clinical scores and composite indicators (58.8%, 20/34). Details of the risk factors identified in each study are presented in Table 3.

**Table 3:** Predictors of extubation failure

**Predictors**	**Alaa et al**	**Bahgat et al**	**Bhat et al**	**Chawla et al**	**Chawla et al**	**Chen et al**	**Cheng et al**	**Dassios et al**	**Devadas et al**	**Dimitriou et al**	**Dimitriou et al**	**Dursun et al**	**Gupta et al**	**Hathlol et al**	**He et al**	**Hermeto et al**	**Hilal et al**	**Hiremath et al**
**Intrinsic factors**																		
Gestational age			**X**							**X**			**X**		**X**	**X**	**X**	
Age at extubation										**X**			**X**		**X**			
Birth weight			**X**						**X**					**X**				
Sex																		
SGA					**X**													
**Maternal factors**																		
Mode of delivery	**X**																	
PROM																		
Chorioamnionitis																		
Antenatal steroids																		
**Diseases and adverse conditions of the newborn**																		
Sepsis							**X**		**X**			**X**						**X**
Anemia							**X**		**X**									**X**
Pneumonia																		**X**
NEC	**X**																	
Severe RDS																		
Arterial hypotension																		
**Treatment of the newborn**																		
Inotropic use														**X**				
≥ 2 doses of surfactant														**X**				
Caffeine administration							**X**											
Unsuccessful enteral feeding																		
**Characteristics before and after extubation**																		
Pre-extubation FiO_2_					**X**								**X**		**X**			
Pre-extubation pH					**X**		**X**						**X**		**X**			
Pre-extubation PCO_2_					**X**										**X**			
Mechanical ventilation duration									**X**									
Mean airway pressure						**X**												
Inspiratory pressure						**X**												
Post-extubation pH																		
Metabolic acidosis in the first 3 days of life																		
Peak FiO_2_ in the first 24 h of age					**X**													
Post-extubation HCO_3_ < 18 mmol/L																		
Expiratory tidal volumes																		
**Clinical scores and composite indicators**																		
RSS(RSS/kg)						**X**						**X**	**X**					
SBT(Δ τ)				**X**				**X**										
Apgar score					**X**	**X**	**X**											
TTdi			**X**															
TTmus			**X**															
PTIdi											**X**							
PTImus											**X**							
LUSS																		
HRV																		
RV																		
Edi																		
NVE																		
Diaphragmatic thickness and excursion		**X**																

**Predictors**	**Hunt et al**	**Kaczmarek et al**	**Kaczmarek et al**	**Kamlin et al**	**Kidman et al**	**Manley et al**	**Menshykov et al**	**Mhanna et al**	**Mohsen et al**	**Ohnstad et al**	**Shalish et al**	**Spaggiari et al**	**Su et al**	**Teixeira et al**	**Wang et al**	**Williams et al**	**Number of studies**
**Intrinsic factors**																	16
Gestational age	**X**				**X**	**X**				**X**		**X**					11
Age at extubation	**X**									**X**						**X**	6
Birth weight	**X**													**X**			5
Sex										**X**							1
SGA																	1
**Maternal factors**																	4
Mode of delivery														**X**			2
PROM												**X**					1
Chorioamnionitis														**X**			1
Antenatal steroids							**X**										1
**Diseases and adverse conditions of the newborn**																	6
Sepsis																	4
Anemia																	3
Pneumonia																	1
NEC																	1
Severe RDS							**X**										1
Arterial hypotension							**X**										1
**Treatment of the newborn**																	3
Inotropic use													**X**				2
≥ 2 doses of surfactant																	1
Caffeine administration																	1
Unsuccessful enteral feeding													**X**				1
**Characteristics before and after extubation**																	13
Pre-extubation FiO_2_	**X**									**X**							5
Pre-extubation pH																	4
Pre-extubation PCO_2_						**X**											3
Mechanical ventilation duration														**X**			2
Mean airway pressure					**X**												2
Inspiratory pressure																	1
Post-extubation pH															**X**		1
Metabolic acidosis in the first 3 days of life							**X**										1
Peak FiO_2_ in the first 24 h of age																	1
Post-extubation HCO_3_ < 18 mmol/L															**X**		1
Expiratory tidal volumes	**X**																1
**Clinical scores and composite indicators**																	20
RSS(RSS/kg)								**X**					**X**				5
SBT(Δ τ)			**X**	**X**							**X**						5
Apgar score										**X**							4
TTdi																	1
TTmus																	1
PTIdi																	1
PTImus																	1
LUSS									**X**								1
HRV		**X**															1
RV			**X**														1
Edi																**X**	1
NVE	**X**																1
Diaphragmatic thickness and excursion																	1

Five variables were found to be definite predictors for EF, based on either all low and moderate risk of bias studies showing a positive association (if at least three studies) or the majority of low and moderate risk of bias studies showing a positive association (if at least five studies). Definite predictors included being of gestational age, sepsis, pre-extubation pH, pre-extubation fraction of inspired oxygen (FiO_2_), and respiratory severity score (RSS). Eight variables were considered likely associated with EF, and these included being of age at extubation, anemia, inotropic use, mean airway pressure (MAP), pre-extubation PCO_2_, mechanical ventilation duration, Apgar score, and spontaneous breathing trial (SBT). 22 variables that showed conflicting results in studies with low and moderate risk of bias, or were positive in only one study, that were considered to have an unclear association with EF, included: birth weight, sex, small for gestational age (SGA), mode of delivery, premature rupture of membranes (PROM), maternal chorioamnionitis, antenatal steroids, pneumonia, necrotizing enterocolitis (NEC), severe respiratory distress syndrome (RDS), arterial hypotension in the first 3 days of life, administration of ≥ 2 doses of surfactant, caffeine administration, unsuccessful enteral feeding, ventilator inspiratory pressure, post-extubation pH < 7.3, metabolic acidosis with pH < 7.25 in the first 3 days of life, peak FiO_2_ within the first 24 h of age, post-extubation HCO_3_ < 18 mmol/L, lung ultrasound severity score (LUSS), heart rate variability (HRV) and electrical activity of the diaphragm (Edi).

**Fig. 3:**
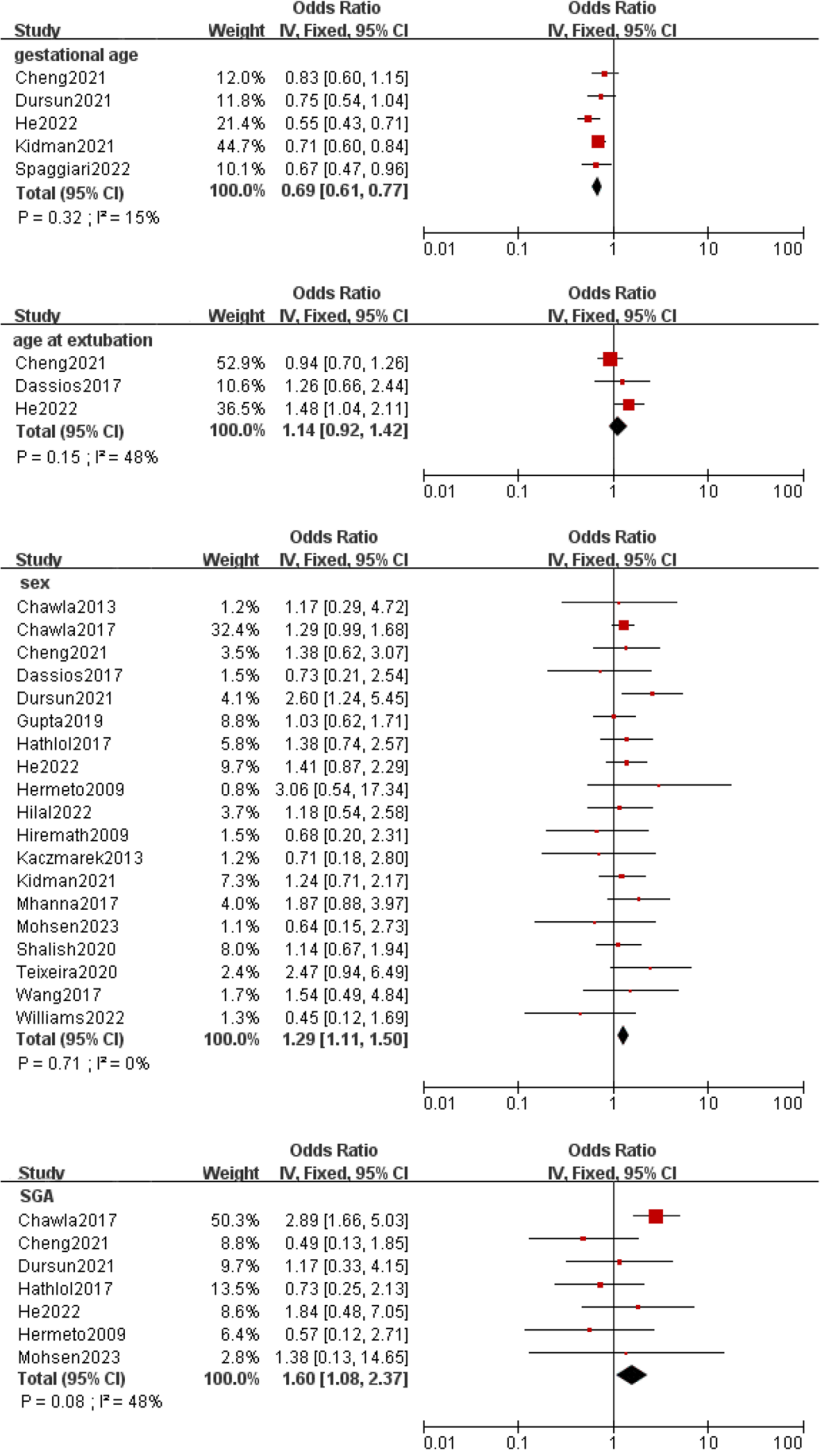
Meta-analysis of intrinsic factors. Forest plots of odds ratios (ORs) that were included in the quantitative meta-analysis and the associated overall ORs. For each OR, the size of the red square region is proportional to the corresponding study weight. Diamond shapes intervals represent the overall ORs. I^2^ represents the fraction of variability among the individual ORs that cannot be explained by sampling variability

**Fig. 4:**
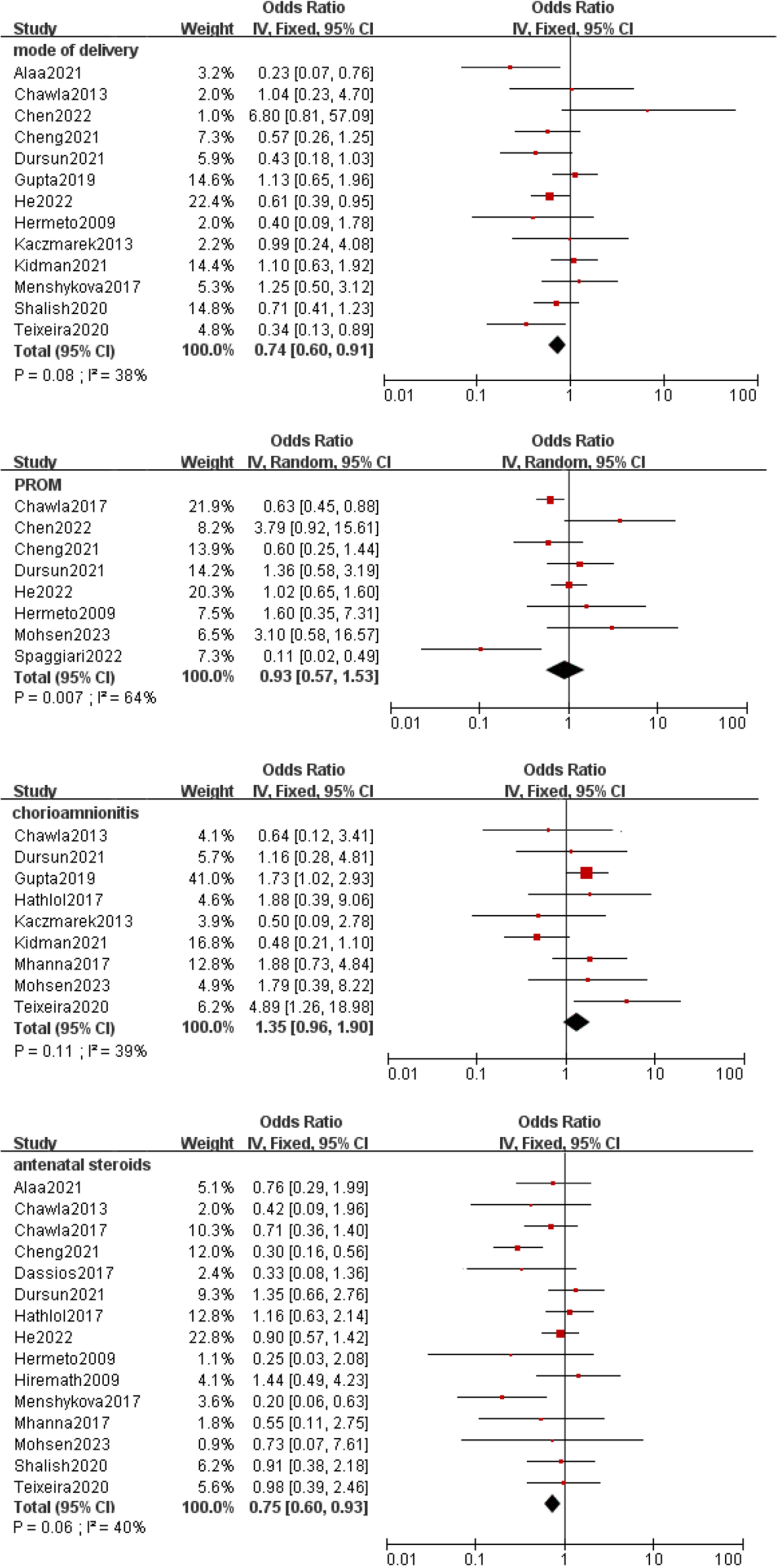
Meta-analysis of maternal factors

**Fig. 5:**
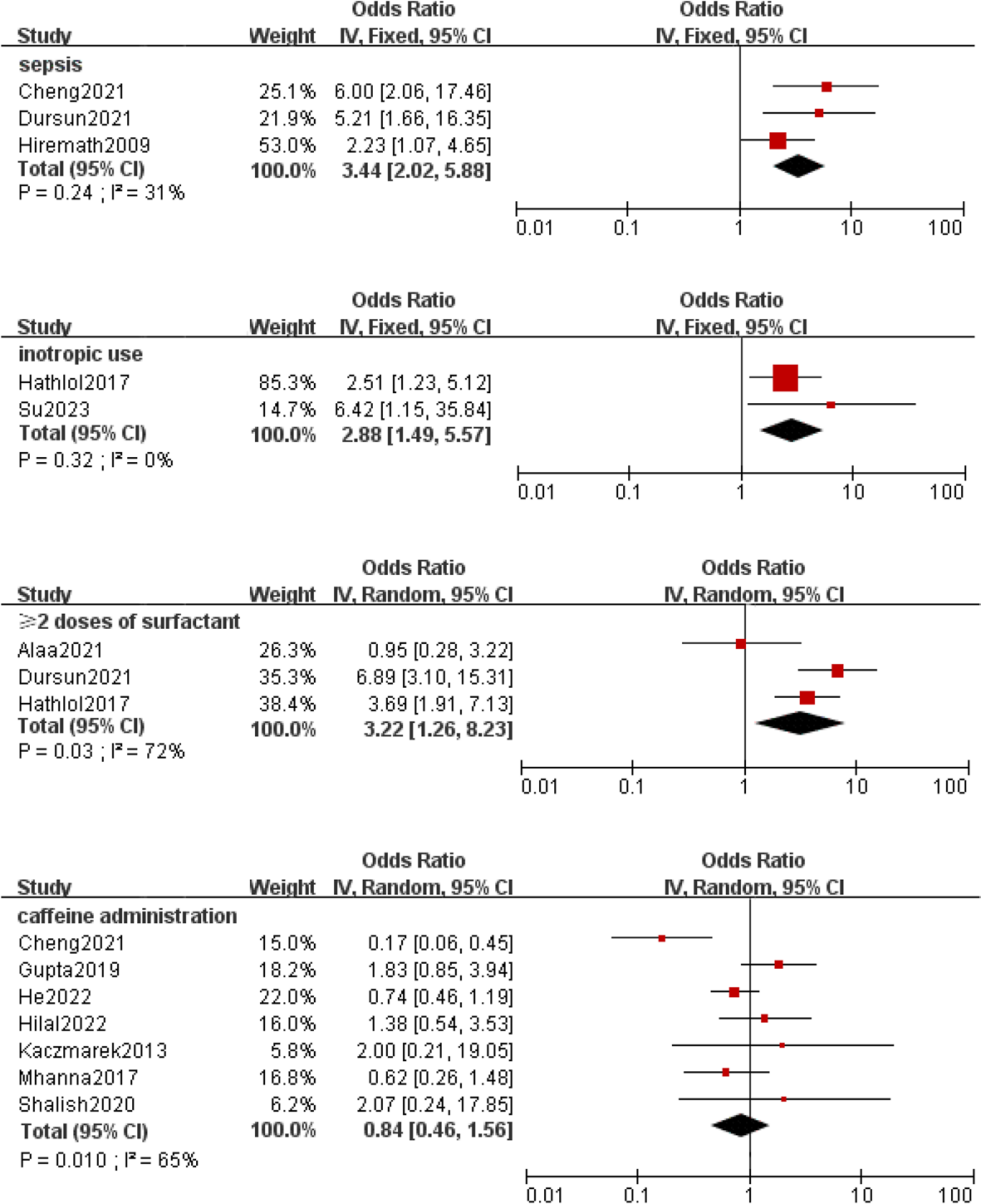
Meta-analysis of disease and treatment factors

**Fig. 6:**
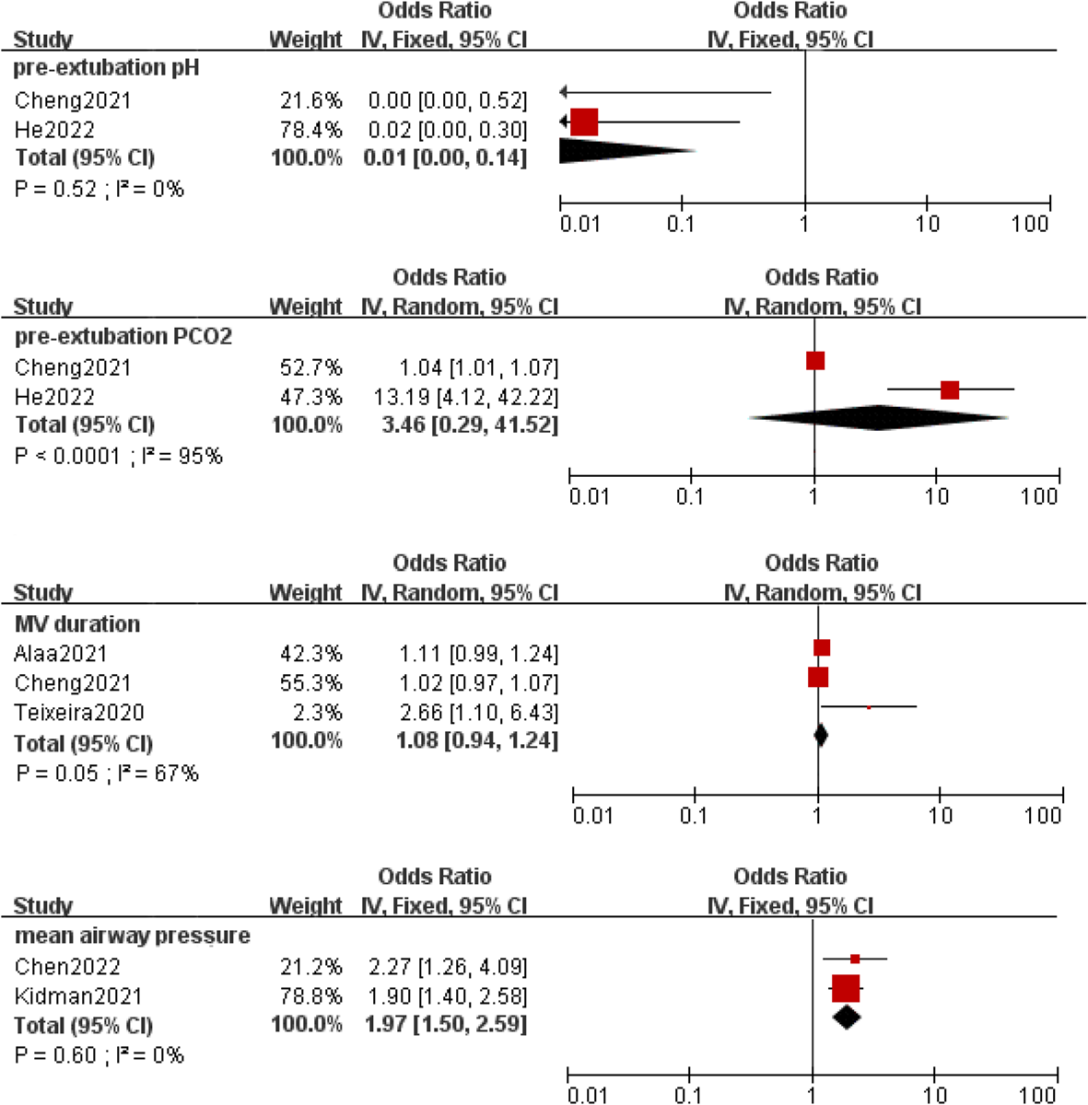
Meta-analysis of characteristics before and after extubation

**Fig. 7:**
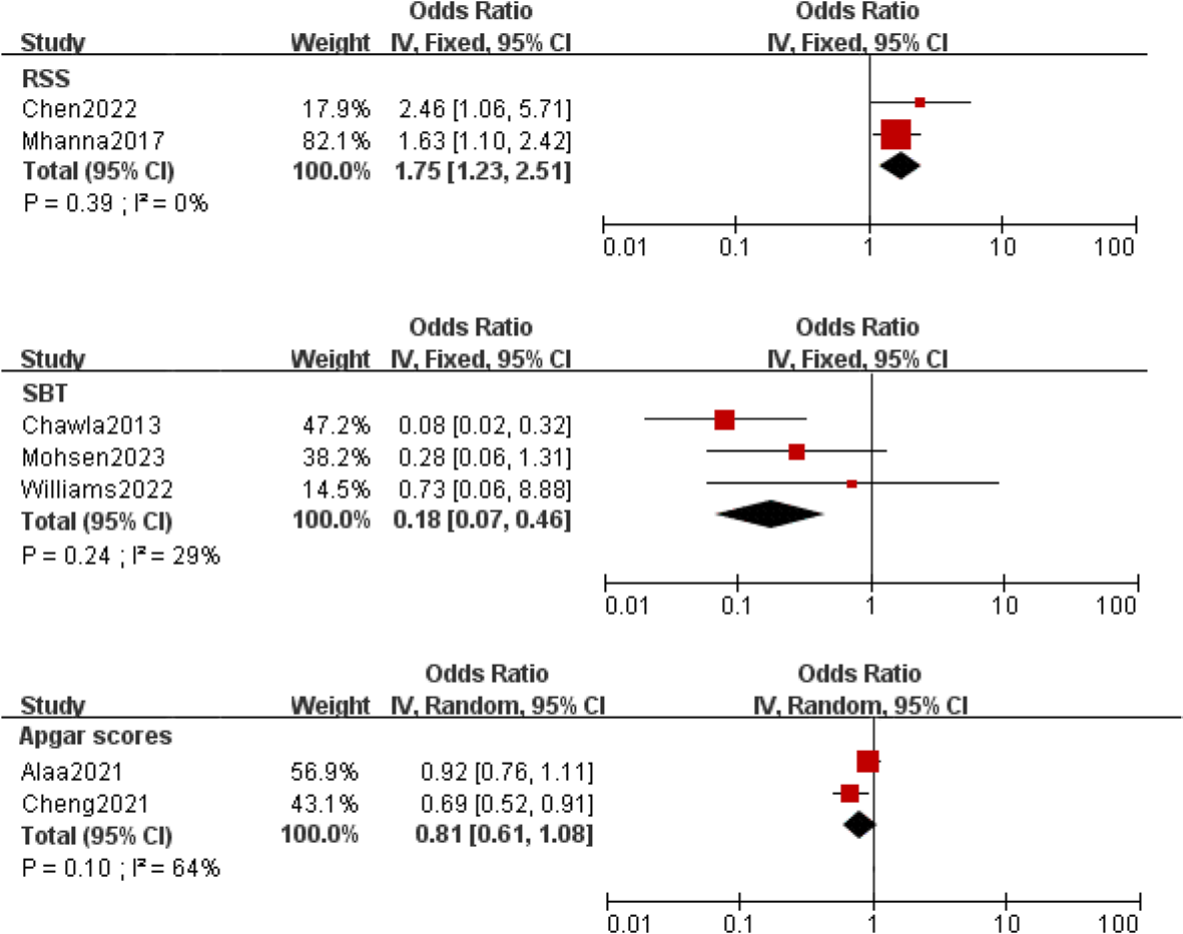
Meta-analysis of clinical scores and composite indicators

Meta-analysis was implemented for 19 predictors with at least two low or moderate risk of bias studies demonstrating homogeneous predictors definitions and reference ranges (Figs. 3, 4, 5, 6, 7).

## Discussion

Our study was the first systematic review of predictors for EF of newborns. Through a qualitative synthesis of 43 predictors and a quantitative meta-analysis of 19 factors from the 34 studies included. We identified five definite factors, eight possible factors, and 22 unclear factors related to EF. Definite factors included gestational age, sepsis, pre-extubation pH, pre-extubation FiO_2_, and RSS. Possible factors included age at extubation, anemia, inotropic use, MAP, pre-extubation PCO_2_, mechanical ventilation duration, Apgar score, and SBT. The results of our systematic review provide an up-to-date comprehensive summary of the latest evidence, which can inform the determination of the optimal timing of extubation in newborns who are mechanically ventilated and the development of interventions to reduce and prevent EF.

Extubation failure reflects a complex pathophysiological process in which multiple factors are implicated such as weak respiratory drive, imbalance between the capacity of the respiratory muscles and the loads imposed on them (lung and chest wall elastic loads, airway and tissue resistance), and inability to keep the airway open. In the last few years, there has been an increasing tendency to extubate intubated newborns early after initial respiratory management. Unfortunately, these fragile infants are often at risk of reintubation shortly after the withdrawal of mechanical ventilation. In our included studies, the reintubation rate was 26.5% (95% CI: 23.1–30.6%) for the first extubation, with up to 40.1% (95% CI: 35.5–44.8%) in VLBW infants. The main causes of reintubation for EF were frequent apnea, increased respiratory workload, and hypoxemia [53]. There is evidence that EF is strongly associated with adverse clinical outcomes and mortality [10, 16, 19, 40, 44].

Our results are consistent with previous findings that gestational age is one of the most critical risk factors for EF. Immature infants are at higher risk for EF than mature infants due to changes in lung maturity, respiratory patterns, and respiratory muscle strength with increasing gestational age. Spaggiari et al. [50] found that the risk of EF is decreased by 27% for every week of gestational age increase. Although another study found no significant association between gestational age and EF, this may be due to differences in the composition of the study population [49]. In China, termination of pregnancy at gestational age of less than 28 weeks is defined as miscarriage in obstetrics. Fetuses younger in gestational age or less viable at birth are frequently more likely to be aborted or abandoned by their parents for treatment. Therefore, the younger the gestational age of the fetus, the less likely it is that the fetus will be transferred to the NICU and receive mechanical ventilation. The development of the brain is critical for the control and regulation of breathing, and a study by Williams et al. [38] showed that higher age at extubation was strongly associated with extubation success (ES) due to a more mature brain. This contradicts the findings of Dimitriou et al. [27] and He et al. [43], who suggested that prolonged ventilation before extubation causes disuse atrophy of the diaphragm, resulting in subsequent EF. Moreover, male infants are more susceptible to EF than female infants [39], and the incidence of meta integration is higher. However, there are no studies to explain the causes of this phenomenon.

Several maternal characteristics affect newborn conditions and outcomes, including mode of delivery, PROM, and maternal infections. Alaa et al. [46] and Teixeira et al. [32] reported that vaginal delivery was significantly associated with EF, although the precise mechanism of this association remains unclear. One possible explanation is that vaginal delivery may occur without the necessary medical care, which could worsen the infant's clinical condition. Furthermore, vaginal delivery itself is in most cases a spontaneous preterm birth due to an underlying inflammatory or infectious disease. Cesarean delivery may be associated with better prenatal care, which may moderate the risk factors associated with preterm birth, thereby reducing the risk of complications and increasing the chances of survival of preterm infants. The impact of PROM on extraction outcomes is also controversial. In 40–70% of patients of low gestational age, PROM is associated with histological chorioamnionitis [54, 55]. Some studies argue that chorioamnionitis may lead to early lung maturation by increasing surface-active substances and reducing pro-inflammatory mediators in the airways [56]. However, we are currently unable to confirm a link between chorioamnionitis and ES. Further studies are required to evaluate whether PROM, independently of chorioamnionitis, can activate unidentified mechanisms that affect lung maturation.

Neonatal diseases and adverse conditions are not only major causes of prolonged hospitalization or even death of newborns but may also be important risk factors for EF. Infection is one of the most common adverse events in hospitalized newborns and poses a threat to all newborns [57]. In our study, sepsis was a common infection affecting EF. First, inflammatory factors can attack immature lung tissue in an inflammatory storm. Once alveolar cells and interstitial lung tissue are destroyed by inflammation, ventilation function, and pulmonary vascular hemodynamics are compromised, and this damage may be irreversible. Second, sepsis may be complicated by encephalitis, leading to central respiratory dysfunction. In addition, three studies reported anemia as a predictor of EF [24, 28, 49]. The possible explanation is that low hemoglobin concentration (HB) levels reduce the oxygen delivery of the respiratory center of the brain and the ability of the lungs to deliver oxygen to tissues, which may lead to an increase in the burden of the heart and lungs, leading to EF.

A systematic review [58] of interventions to improve ES in neonates showed that methylxanthines improved rates of ES and caffeine given pre-extubation reduced time spent in oxygen and rates of death or disability. However, caffeine was routinely used in most studies we included, so no significant differences were found. There is no unified standard for the specific dosage and duration of administration, and further studies are needed. Two studies [40, 48] showed a significant association between the use of inotropes and EF. Early hypotension or blood pressure instability has previously been documented to impact lung and brain development, while it is unclear through which mechanisms the use of inotropes in early life affects EF [17]. In addition, the early introduction of total enteral feeding may affect extubation outcomes by enhancing micronutrient delivery, promoting gut development and maturation, stimulating microbiome development, reducing inflammation, and enhancing brain growth and neurodevelopment [48, 59].

Currently, the decision to extubate relies on clinical judgment through the interpretation of ventilatory support, blood gas values, and overall clinical stability of the neonate [37]. Pre-extubation pH, pre-extubation FiO_2_, pre-extubation PCO_2_, and MAP are important markers of extubation readiness and significant predictors of EF [16, 17, 19, 31, 39, 41–43, 49]. A lower pH indicates that the oxygen exchange capacity of the lungs is not meeting the body's demand for oxygen supply. Mechanically ventilated infants with severe hypercapnia are unlikely to produce sufficient spontaneous tidal volume for ES. Since blood gas data before extubation are highly dependent on ventilator settings. Wang et al. [51] found that postextraction arterial blood gas analysis results were more valuable in predicting EF than pre-extubation data. However, accurate thresholds for the above predictors are currently lacking and they require additional confirmation in multicenter studies with high sample sizes. In addition, long-term mechanical ventilation may cause damage to respiratory muscle strength and neural development [5]. Spaggiari et al. [50] pointed out that for every additional day of mechanical ventilation, EF increases by 5%, which is similar to the research results of Devadas et al. [24]. In the past decade, the practice of prompt weaning and early extubation to non-invasive respiratory support has been the focus and ultimate goal. Continuous positive airway pressure (CPAP) is the most commonly used respiratory support in clinical practice. The latest Cochrane Systematic review shows [60] that nasal intermittent positive pressure ventilation (NIPPV) is more effective than nasal CPAP in reducing the incidence of EF and the need for reintubation within 48 h to one week, but does not significantly reduce chronic lung disease and mortality. Noninvasive high-frequency oscillatory ventilation (NHFOV) is an unconventional noninvasive ventilation mode that is considered a possible improvement over CPAP. However, there is still a lack of uniform standards and consensus on which noninvasive modality to use for respiratory support after extubation.

ES depends on the adequacy of respiratory drive, the capacity of the respiratory muscles, and the load imposed upon them. Given this, EF is more likely to be predicted by a composite evaluation than a univariate index. RSS is a simple, non-invasive, easy-to-use tool for assessing respiratory failure. It has been effectively used to indicate the severity of lung disease in several large multicenter studies [61, 62], with five studies showing that high RSS or RSS/kg values before extubation strongly correlate with EF [10, 41, 42, 47, 48]. The low Apgar score indicates that early neonatal hypoxia may be prolonged, and cause significant hypoxic damage to the brain and lungs, affecting the recovery of respiratory function, which may lead to difficulty in extubation and prolong the use of the ventilator [17, 39, 41, 49]. The SBT was developed for the adult population to assess the patient's ability to breathe spontaneously with minimal or no support. Incorporating SBT into weaning protocols is an accepted common practice in adult populations [63]. Despite the widespread use of SBT in neonatal intensive care units (NICUs) worldwide, few robust studies have been conducted in neonatal populations. A systematic review evaluating the accuracy of all extraction preparation tests in preterm infants, including SBT, concluded that there is insufficient evidence to support using SBT in preterm infants [64]. Additionally, loss of variability is a common occurrence in disease and is often predictive of poor outcomes. Heart rate variability and respiratory variability are two attractive tools, they are simple and noninvasive to measure and can be automated and performed at the bedside [23, 52]. However, the predictive value of variability needs to be tested in a larger population and through a randomized controlled trial design.

The strengths of this systematic review include the systematic approach to identifying all publications that included predictors for EF of newborns and the division of predictors into six major categories to provide a logical progression of possible factors of EF. However, The results of this systematic review and meta-analysis must be considered in the context of several limitations. First, the lack of standardization in the definition of EF. In addition, while the search strategy was comprehensive and rigorous, it may still have missed some studies. Finally, because most of the included studies were retrospective, causal assertions could not be made regarding the predictors for EF.

## Conclusions

In summary, we identified several of the most critical factors affecting extubation in our published studies, including gestational age, sepsis, pre-extubation pH, pre-extubation FiO_2_, and RSS. However, all of the included studies did not take into account how sociodemographic factors, such as family income, and the mental and physical health of the parents can affect EF. In addition to this, the level of NICU team skills, antenatal and delivery room management may impact both severity of illness and extubation outcome. Therefore, well-designed and more extensive prospective studies investigating the predictors affecting EF are still needed in the future. Additionally, consensus on the definition of EF is needed to better compare results and to improve the reliability of meta-analyses. In recent years, machine learning (ML) methods have been increasingly applied to handle a variety of challenging medical issues. ML can help improve the reliability, performance, predictability, and accuracy of diagnostic systems for many diseases. Since many confounding factors affect extubation outcomes, future research should explore the possibility of combining various tools. It can develop predictive models based on ML to predict EF more robustly.

### Supplementary Information

**Additional file 1:** **Supplemental Digital.** Database search strings. **Fig. S1.** Meta integration of EF rate. **Fig. S2.** Meta integration of EF rate in female infants. **Fig. S3.** Meta integration of EF rate in male infants. **Fig. S4.** Meta integration of EF rate in VLBW infants. **Fig. S5.** Meta integration of EF rate in extremely preterm infants. **Fig. S6.** Meta integration of EF rate for different extubation criteria. **Fig. S7.** Quality evaluation of each study.

## Data Availability

The datasets used and/or analysed during the current study are available from the corresponding author on reasonable request.

## Abbreviations

MV: Mechanical ventilation
EF: Extubation failure
FiO_2_: Inspired oxygen fraction
PCO_2_: Partial pressure of carbon dioxide
NCPAP: Nasal continuous positive airway pressure
NIPPV: Nasal intermittent positive pressure ventilation
NC: Nasal cannula
HFNC: High-flow nasal cannula
NIV: Non-invasive ventilation
PS: Pulmonary surfactant
NHF: Nasal high-flow therapy
HIE: Hypoxic-ischemic encephalopathy
IVH: Intraventricular hemorrhage
NIMV: Nasal intermittent mandatory ventilation
BiPAP: Bilevel positive airway pressure
SARS: Silverman anderson retraction score
PMA: Postmenstrual age
SGA: Small for gestational age
PROM: Premature rupture of membranes
NEC: Necrotizing enterocolitis
RDS: Respiratory distress syndrome
RSS: Respiratory severity score
SBT: Spontaneous breathing trial
TTdi: Tension-time index of the diaphragm
TTmus: Tension-time index of the respiratory muscles
PTIdi: Diaphragmatic pressure–time index
PTImus: Pressure–time index of the respiratory muscles
LUSS: Lung ultrasound severity score
HRV: Heart rate variability
RV: Respiratory variability
Edi: Electrical activity of the diaphragm
NVE: Neuroventilatory efficiency
