# Supplementary material for: Predictors of extubation failure in newborns: a systematic review and meta-analysis
Source: Ital J Pediatr. 2023 Oct 2;49:133. doi: 10.1186/s13052-023-01538-0 (PMC10546653; doi:10.1186/s13052-023-01538-0)
Supplement: Supplementary file 1 — Additional file 1: Supplemental Digital. Database search strings. Fig. S1. Meta integration of EF rate. Fig. S2. Meta integration of EF rate in female infants. Fig. S3. Meta integration of EF rate in male infants. Fig. S4. Meta integration of EF rate in VLBW infants. Fig. S5. Meta integration of EF rate in extremely preterm infants. Fig. S6. Meta integration of EF rate for different extubation criteria. Fig. S7. Quality evaluation of each study. [file 13052_2023_1538_MOESM1_ESM.docx]

**Supplemental Digital: Database search strings**

**Pubmed**

**((("Airway Extubation"[Mesh]) OR ((((((((((((((("Extubation failure"[Title/Abstract]) ) OR ("Extubation outcome"[Title/Abstract])) OR ("Successful extubation"[Title/Abstract])) OR ("extubation success"[Title/Abstract])) OR ("extubation readiness"[Title/Abstract])) OR (extubat*[Title/Abstract])) OR ("Extubation, Airway"[Title/Abstract])) OR ("Tracheal Extubation"[Title/Abstract])) OR ("Extubation, Tracheal"[Title/Abstract])) OR ("Tracheal Extubations"[Title/Abstract])) OR ("Intratracheal Extubation"[Title/Abstract])) OR ("Endotracheal Extubation"[Title/Abstract])) OR ("Endotracheal Extubations"[Title/Abstract])) OR ("Extubation, Endotracheal"[Title/Abstract]))) AND ((((((("Infant, Newborn"[Mesh]) OR ("Infant, Premature"[Mesh])) OR ("Infant, Extremely Premature"[Mesh])) OR ("Infant, Low Birth Weight"[Mesh])) OR ("Infant, Extremely Low Birth Weight"[Mesh])) OR ("Infant, Very Low Birth Weight"[Mesh])) OR ((((((((((((((((((((((((((((((((((((((((((("Infants, Newborn"[Title/Abstract]) ) OR ("Newborn Infant"[Title/Abstract])) OR ("Newborn Infants "[Title/Abstract])) OR (Newborns[Title/Abstract])) OR (Newborn[Title/Abstract])) OR (Neonate[Title/Abstract])) OR (Neonates[Title/Abstract])) OR ("Infants, Premature"[Title/Abstract])) OR ("Premature Infant"[Title/Abstract])) OR ("Preterm Infants"[Title/Abstract])) OR ("Infant, Preterm"[Title/Abstract])) OR ("Infants, Preterm"[Title/Abstract])) OR ("Preterm Infant"[Title/Abstract])) OR ("Premature Infants"[Title/Abstract])) OR ("Infant, Postmature "[Title/Abstract])) OR ("Infant, Small for Gestational Age "[Title/Abstract])) OR ("Infant, Extremely Premature "[Title/Abstract])) OR ("Extremely Premature Infant"[Title/Abstract])) OR ("Infants, Extremely Premature"[Title/Abstract])) OR ("Premature Infants, Extremely"[Title/Abstract])) OR ("Extremely Preterm Infants"[Title/Abstract])) OR ("Extremely Preterm Infant"[Title/Abstract])) OR ("Infants, Extremely Preterm"[Title/Abstract])) OR ("Preterm Infant, Extremely"[Title/Abstract])) OR ("Preterm Infants, Extremely"[Title/Abstract])) OR ("Extremely Premature Infants"[Title/Abstract])) OR ("Very-Low-Birth-Weight Infant"[Title/Abstract])) OR ("Infant, Very-Low-Birth-Weight"[Title/Abstract])) OR ("Infants, Very-Low-Birth-Weight"[Title/Abstract])) OR ("Very Low Birth Weight Infant"[Title/Abstract])) OR ("Very-Low-Birth-Weight Infants"[Title/Abstract])) OR ("Very Low Birth Weight"[Title/Abstract])) OR ("Low-Birth-Weight Infant"[Title/Abstract])) OR ("Infant, Low-Birth-Weight"[Title/Abstract])) OR ("Infants, Low-Birth-Weight"[Title/Abstract])) OR ("Low Birth Weight Infant"[Title/Abstract])) OR ("Low-Birth-Weight Infants"[Title/Abstract])) OR ("Low Birth Weight"[Title/Abstract])) OR ("Birth Weight, Low"[Title/Abstract])) OR ("Birth Weights, Low"[Title/Abstract])) OR ("Low Birth Weights"[Title/Abstract])) OR ("Extremely Low Birth Weight Infant"[Title/Abstract])))) AND (("Risk Factors"[Mesh]) OR (((((((((((((((determinants[Title/Abstract]) OR ("clinical predictors "[Title/Abstract])) OR (predictors[Title/Abstract])) OR ("Factor, Risk"[Title/Abstract])) OR ("Risk Factor "[Title/Abstract])) OR ("influencing factors"[Title/Abstract])) OR ("influencing factor "[Title/Abstract])) OR (indicators[Title/Abstract])) OR (causality[Title/Abstract])) OR (cause[Title/Abstract])) OR (causes[Title/Abstract])) OR (factors[Title/Abstract])) OR (factor[Title/Abstract])) OR ("Risk Assessment "[Title/Abstract])) OR ("risk assessments"[Title/Abstract])))**

**Web of science**

#1

**((((((((((((((((((((((((((((TS=(“Airway Extubation”)) OR TI=(“Extubation failure”)) OR AB=(“Extubation failure”)) OR TI=(“Extubation outcome”)) OR AB=(“Extubation outcome”)) OR TI=(“Successful extubation”)) OR AB=(“Successful extubation”)) OR TI=(“extubation success”)) OR AB=(“extubation success”)) OR TI=(“extubation readiness”)) OR AB=(“extubation readiness”)) OR TI=(extubat*)) OR AB=(extubat*)) OR TI=(“Extubation, Airway”)) OR AB=(“Extubation, Airway”)) OR TI=(“Tracheal Extubation”)) OR AB=(“Tracheal Extubation”)) OR TI=(“Extubation, Tracheal”)) OR AB=(“Extubation, Tracheal”)) OR TI=(“Tracheal Extubations”)) OR AB=(“Tracheal Extubations”)) OR TI=(“Intratracheal Extubation”)) OR AB=(“Intratracheal Extubation”)) OR TI=(“Endotracheal Extubation”)) OR AB=(“Endotracheal Extubation”)) OR TI=(“Endotracheal Extubations”)) OR AB=(“Endotracheal Extubations”)) OR TI=(“Extubation, Endotracheal”)) OR AB=(“Extubation, Endotracheal”)**

**#2**

**((((((((((((((((((((((((((((((((((((((((((((((((((((((((((((((((((((((((((((((((((((((((TS=(“Infant, Newborn”)) OR TS=(“Infant, Premature”)) OR TS=(“Infant, Extremely Premature”)) OR TS=(“Infant, Very Low Birth Weight”)) OR TS=(“Infant, Extremely Low Birth Weight”)) OR TI=(“Infants, Newborn”)) OR AB=(“Infants, Newborn”)) OR TI=(“Newborn Infant”)) OR AB=(“Newborn Infant”)) OR TI=(“Newborn Infants”)) OR AB=(“Newborn Infants”)) OR TI=(Newborns)) OR AB=(Newborns)) OR TI=(Newborn)) OR AB=(Newborn)) OR TI=(Neonate)) OR AB=(Neonate)) OR TI=(Neonates)) OR AB=(Neonates)) OR TI=(“Infants, Premature”)) OR AB=(“Infants, Premature”)) OR TI=(“Premature Infant”)) OR AB=(“Premature Infant”)) OR TI=(“Preterm Infants”)) OR AB=(“Preterm Infants”)) OR TI=(“Infant, Preterm”)) OR AB=(“Infant, Preterm”)) OR TI=(“Infants, Preterm”)) OR AB=(“Infants, Preterm”)) OR TI=(“Preterm Infant”)) OR AB=(“Preterm Infant”)) OR TI=(“Premature Infants”)) OR AB=(“Premature Infants”)) OR TI=(“Infant, Postmature”)) OR AB=(“Infant, Postmature”)) OR TI=(“Infant, Small for Gestational Age”)) OR AB=(“Infant, Small for Gestational Age”)) OR TI=(“Infant, Extremely Premature”)) OR AB=(“Infant, Extremely Premature”)) OR TI=(“Extremely Premature Infant”)) OR AB=(“Extremely Premature Infant”)) OR TI=(“Infants, Extremely Premature”)) OR AB=(“Infants, Extremely Premature”)) OR TI=(“Premature Infants, Extremely”)) OR AB=("Premature Infants, Extremely")) OR TI=("Extremely Preterm Infants")) OR AB=("Extremely Preterm Infants")) OR TI=("Extremely Preterm Infant")) OR AB=("Extremely Preterm Infant")) OR TI=("Infants, Extremely Preterm")) OR AB=("Infants, Extremely Preterm")) OR TI=("Preterm Infant, Extremely")) OR AB=("Preterm Infant, Extremely")) OR TI=("Preterm Infants, Extremely")) OR AB=("Preterm Infants, Extremely")) OR TI=("Extremely Premature Infants")) OR AB=("Extremely Premature Infants")) OR TI=("Very-Low-Birth-Weight Infant")) OR AB=("Very-Low-Birth-Weight Infant")) OR TI=("Infant, Very-Low-Birth-Weight")) OR AB=("Infant, Very-Low-Birth-Weight")) OR TI=("Infants, Very-Low-Birth-Weight")) OR AB=("Infants, Very-Low-Birth-Weight")) OR TI=("Very Low Birth Weight Infant")) OR AB=("Very Low Birth Weight Infant")) OR TI=("Very-Low-Birth-Weight Infants")) OR AB=("Very-Low-Birth-Weight Infants")) OR TI=("Very Low Birth Weight")) OR AB=("Very Low Birth Weight")) OR TI=("Low-Birth-Weight Infant")) OR AB=("Low-Birth-Weight Infant")) OR TI=("Infant, Low-Birth-Weight")) OR AB=("Infant, Low-Birth-Weight")) OR TI=("Infants, Low-Birth-Weight")) OR AB=("Infants, Low-Birth-Weight")) OR TI=("Low Birth Weight Infant")) OR AB=("Low Birth Weight Infant")) OR TI=("Low-Birth-Weight Infants")) OR AB=("Low-Birth-Weight Infants")) OR TI=("Low Birth Weight")) OR AB=("Low Birth Weight")) OR TI=("Birth Weight, Low")) OR AB=("Birth Weight, Low")) OR TI=("Birth Weights, Low")) OR AB=("Birth Weights, Low")) OR TI=("Low Birth Weights")) OR AB=("Low Birth Weights")) OR TI=("Extremely Low Birth Weight Infant")) OR AB=("Extremely Low Birth Weight Infant")**

**#3**

**((((((((((((((((((((((((((((((TS=("risk factors")) OR TI=(determinants)) OR AB=(determinants )) OR TI=("clinical predictors")) OR AB=("clinical predictors")) OR TI=(predictors)) OR AB=(predictors)) OR TI=("Factor, Risk")) OR AB=("Factor, Risk")) OR TI=("Risk Factor")) OR AB=("Risk Factor")) OR TI=("influencing factors")) OR AB=("influencing factors")) OR TI=("influencing factor")) OR AB=("influencing factor")) OR TI=(indicators)) OR AB=(indicators)) OR TI=(causality)) OR AB=(causality)) OR TI=(cause)) OR AB=(cause)) OR TI=(causes)) OR AB=(causes)) OR TI=(factors)) OR AB=(factors)) OR TI=(factor)) OR AB=(factor)) OR TI=("Risk Assessment")) OR AB=("Risk Assessment")) OR TI=("risk assessments")) OR AB=("risk assessments")**

**#4**

**#1 and #2 and #3**

**Embase**

#1 'extubation'/exp

#2 'airway extubation':ti,ab,kw OR 'extubation failure':ti,ab,kw OR 'extubation outcome':ti,ab,kw OR 'successful extubation':ti,ab,kw OR 'extubation success':ti,ab,kw OR 'extubation readiness':ti,ab,kw OR extubat*:ti,ab,kw OR 'extubation, airway':ti,ab,kw OR 'tracheal extubation':ti,ab,kw OR 'extubation, tracheal':ti,ab,kw OR 'tracheal extubations':ti,ab,kw OR 'intratracheal extubation':ti,ab,kw OR 'endotracheal extubation':ti,ab,kw OR 'endotracheal extubations':ti,ab,kw OR 'extubation, endotracheal':ti,ab,kw

#3 #1 OR #2

#4 'newborn'/exp

#5 'prematurity'/exp

#6 'very low birth weight'/exp

#7 'extremely low birth weight'/exp

#8 'low birth weight'/exp

#9 'infant, newborn':ti,ab,kw OR 'infant, premature':ti,ab,kw OR 'infant, very low birth weight':ti,ab,kw OR 'infant, low birth weight':ti,ab,kw OR 'infant, extremely low birth weight':ti,ab,kw OR 'infants, newborn':ti,ab,kw OR 'newborn infant':ti,ab,kw OR 'newborn infants':ti,ab,kw OR newborns:ti,ab,kw OR newborn:ti,ab,kw OR neonate:ti,ab,kw OR neonates:ti,ab,kw OR 'infants, premature':ti,ab,kw OR 'premature infant':ti,ab,kw OR 'preterm infants':ti,ab,kw OR 'infant, preterm':ti,ab,kw OR 'infants, preterm':ti,ab,kw OR 'preterm infant':ti,ab,kw OR 'premature infants':ti,ab,kw OR 'infant, postmature':ti,ab,kw OR 'small for gestational age':ti,ab,kw OR 'infant, extremely premature':ti,ab,kw OR 'extremely premature infant':ti,ab,kw OR 'infants, extremely premature':ti,ab,kw OR 'premature infants, extremely':ti,ab,kw OR 'extremely preterm infants':ti,ab,kw OR 'extremely preterm infant':ti,ab,kw OR 'infants, extremely preterm':ti,ab,kw OR 'preterm infant, extremely':ti,ab,kw OR 'preterm infants, extremely':ti,ab,kw OR 'extremely premature infants':ti,ab,kw OR 'very-low-birth-weight infant':ti,ab,kw OR 'infant, very-low-birth-weight':ti,ab,kw OR 'infants, very-low-birth-weight':ti,ab,kw OR 'very low birth weight infant':ti,ab,kw OR 'very-low-birth-weight infants':ti,ab,kw OR 'very low birth weight':ti,ab,kw OR 'low-birth-weight infant':ti,ab,kw OR 'infant, low-birth-weight':ti,ab,kw OR 'infants, low-birth-weight':ti,ab,kw OR 'low birth weight infant':ti,ab,kw OR 'low-birth-weight infants':ti,ab,kw OR 'low birth weight':ti,ab,kw OR 'birth weight, low':ti,ab,kw OR 'birth weights, low':ti,ab,kw OR 'low birth weights':ti,ab,kw OR 'extremely low birth weight infant':ti,ab,kw

#10 #4 OR #5 OR #6 OR #7 OR #8 OR #9

#11 'risk factor'/exp

#12 determinants:ti,ab,kw OR 'clinical predictors':ti,ab,kw OR predictors:ti,ab,kw OR 'factor, risk':ti,ab,kw OR 'risk factor':ti,ab,kw OR 'influencing factors':ti,ab,kw OR 'influencing factor':ti,ab,kw OR indicators:ti,ab,kw OR causality:ti,ab,kw OR 'risk assessments':ti,ab,kw OR 'risk assessment':ti,ab,kw OR factor:ti,ab,kw OR factors:ti,ab,kw OR causes:ti,ab,kw OR cause:ti,ab,kw

#13 #11 OR #12

#14 #3 AND #10 AND #13

**Cochrane**

#1 MeSH descriptor: [Airway Extubation] explode all trees

#2 (“Extubation failure” OR “Extubation outcome” OR “Successful extubation” OR “extubation success” OR “extubation readiness” OR extubat* OR “Extubation, Airway” OR “Tracheal Extubation” OR “Extubation, Tracheal” OR “Tracheal Extubations” OR “Intratracheal Extubation” OR “Endotracheal Extubation” OR “Endotracheal Extubations” OR “Extubation, Endotracheal”):ti,ab,kw

#3 #1 OR #2

#4 MeSH descriptor: [Infant, Newborn] explode all trees

#5MeSH descriptor: [Infant, Premature] explode all trees

#6MeSH descriptor: [Infant, Extremely Premature] explode all trees

#7MeSH descriptor: [Infant, Very Low Birth Weight] explode all trees

#8MeSH descriptor: [Infant, Low Birth Weight] explode all trees

#9MeSH descriptor: [Infant, Extremely Low Birth Weight] explode all trees

#10 (“Infants, Newborn” OR “Newborn Infant” OR “Newborn Infants” OR Newborns OR Newborn OR Neonate OR Neonates OR “Infants, Premature” OR “Premature Infant” OR “Preterm Infants” OR “Infant, Preterm” OR “Infants, Preterm” OR “Preterm Infant” OR “Premature Infants” OR “Infant, Postmature” OR “Infant, Small for Gestational Age” OR “Infant, Extremely Premature” OR “Extremely Premature Infant” OR “Infants, Extremely Premature” OR “Premature Infants, Extremely” OR “Extremely Preterm Infants” OR “Extremely Preterm Infant” OR “Infants, Extremely Preterm” OR “Preterm Infant, Extremely” OR “Preterm Infants, Extremely” OR “Extremely Premature Infants” OR “Very-Low-Birth-Weight Infant” OR “Infant, Very-Low-Birth-Weight” OR “Infants, Very-Low-Birth-Weight” OR “Very Low Birth Weight Infant” OR “Very-Low-Birth-Weight Infants” OR “Very Low Birth Weight” OR “Low-Birth-Weight Infant” OR “Infant, Low-Birth-Weight” OR “Infants, Low-Birth-Weight” OR “Low Birth Weight Infant” OR “Low-Birth-Weight Infants” OR “Low Birth Weight” OR “Birth Weight, Low” OR “Birth Weights, Low” OR “Low Birth Weights” OR “Extremely Low Birth Weight Infant”):ti,ab,kw

#11#4 OR #5 OR #6 OR #7 OR #8 OR #9 OR #10

#12MeSH descriptor: [Risk Factors] explode all trees

#13 (Determinants OR “clinical predictors” OR predictors OR “Factor, Risk” OR “Risk Factor” OR “influencing factors” OR “influencing factor” OR indicators OR causality OR cause OR causes OR factors OR factor OR “Risk Assessment” OR “risk assessments”):ti,ab,kw

#14#12 OR #13

#15#3 AND #11 AND #14

**Supplemental Digital: Fig.S1-S7**

**
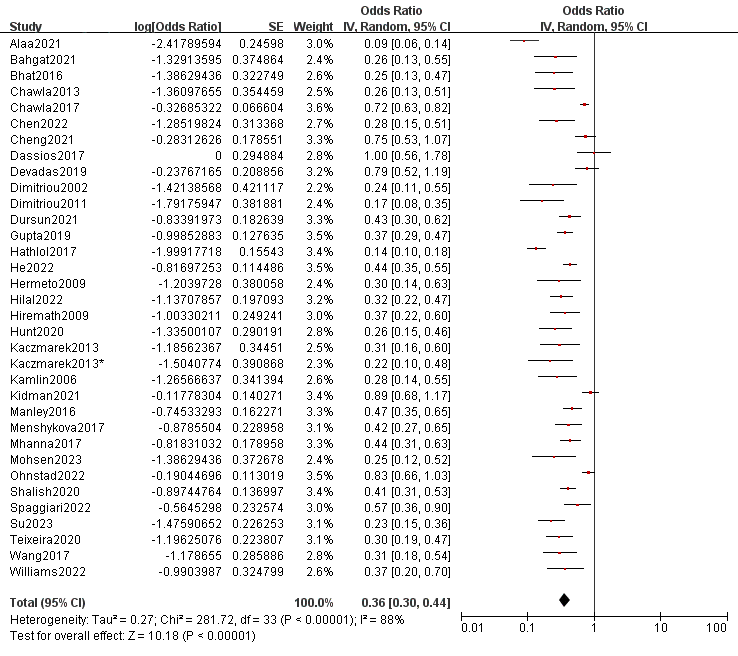
**

Fig. S1 Meta integration of EF rate


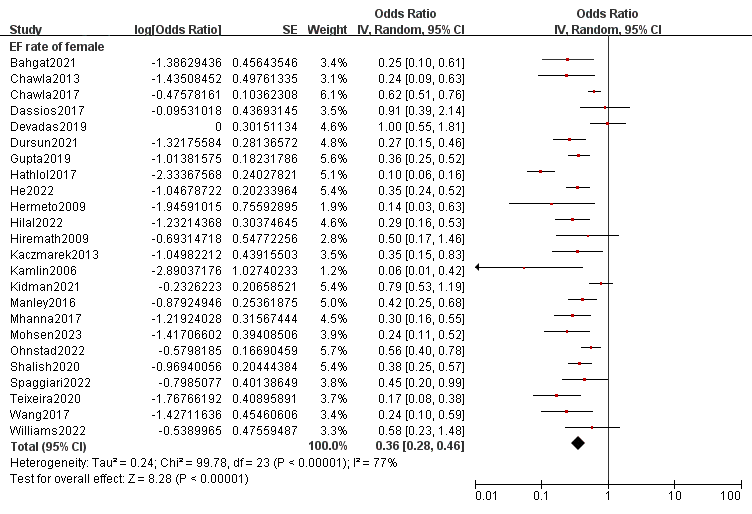


Fig. S2 Meta integration of EF rate in female infants


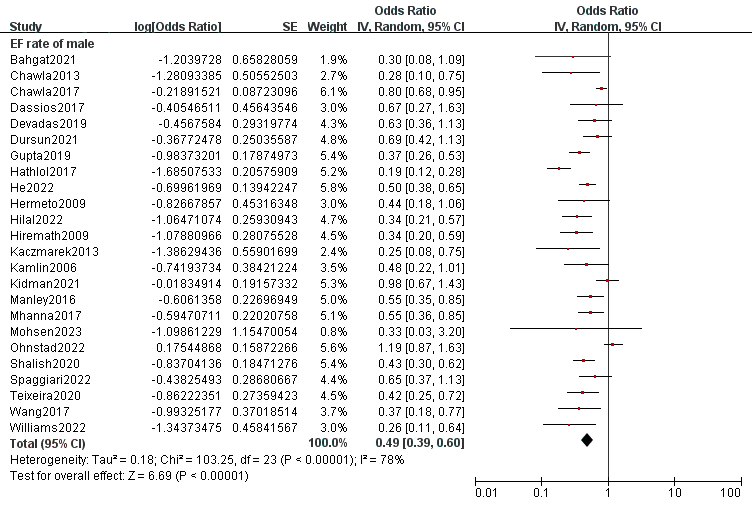


Fig. S3 Meta integration of EF rate in male infants


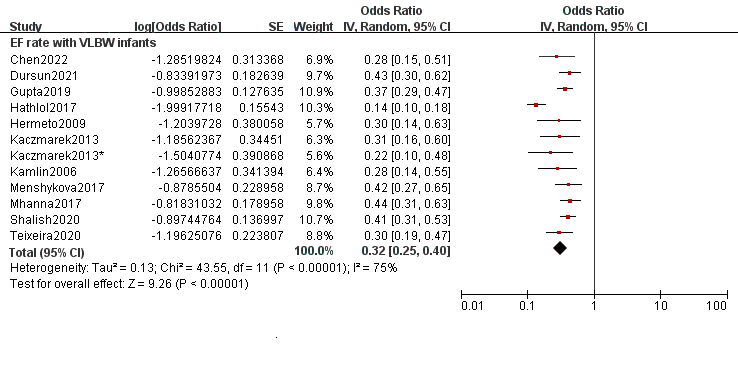


Fig. S4 Meta integration of EF rate in VLBW infants


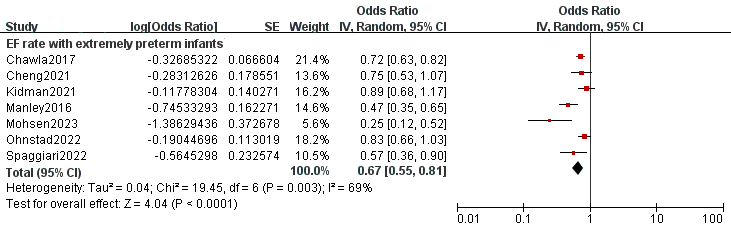


Fig. S5 Meta integration of EF rate in extremely preterm infants


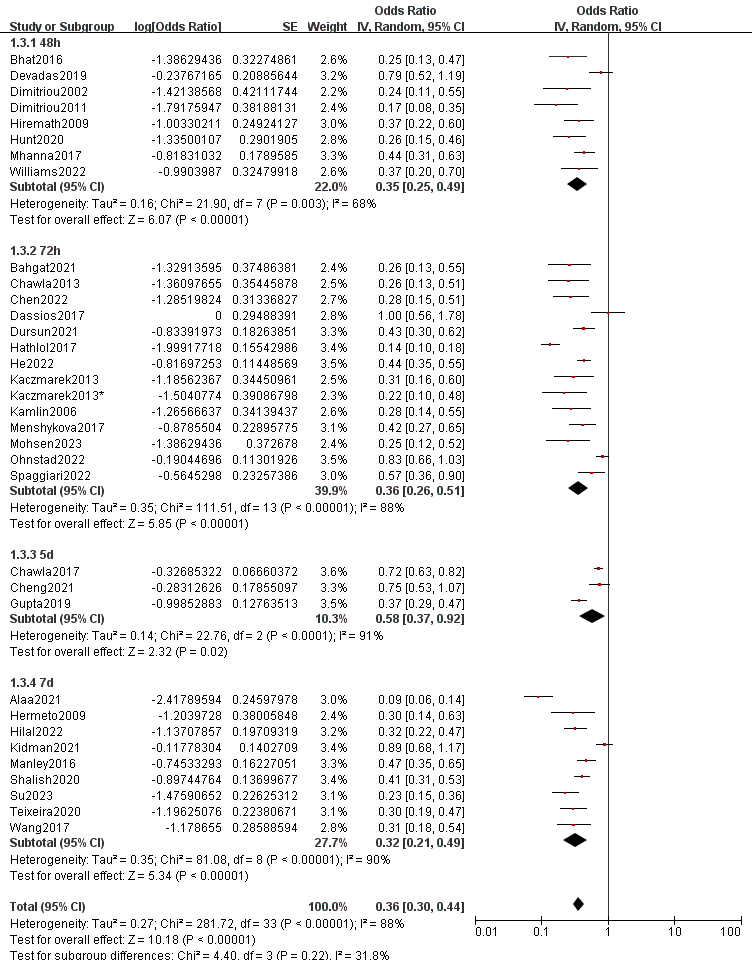


Fig. S6 Meta integration of EF rate for different extubation criteria


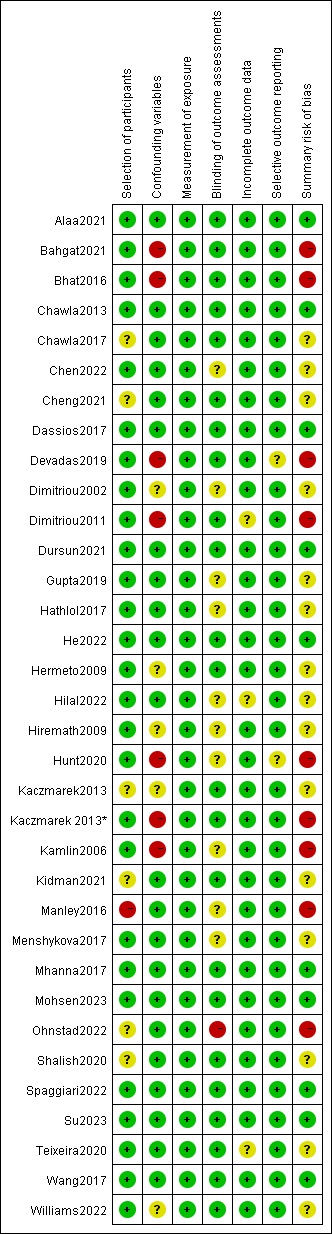


Fig. S7 Quality evaluation of each study
